# The antinociception of oxytocin on colonic hypersensitivity in rats was mediated by inhibition of mast cell degranulation via Ca^2+^-NOS pathway

**DOI:** 10.1038/srep31452

**Published:** 2016-08-19

**Authors:** Liping Gong, Jing Li, Yan Tang, Ting Han, Chuanfei Wei, Xiao Yu, Jingxin Li, Rong Wang, Xuelian Ma, Kejing Liu, Lingyun Geng, Shaozhuang Liu, Bing Yan, Chuanyong Liu

**Affiliations:** 1 Department of Physiology, School of Medicine, Shandong University, China; 2 Central Hospital of Zibo, Zibo, China; 3 Liaocheng People’s Hospital, Liaocheng, China; 4 Shandong Provincial Hospital Affiliated to Shandong University, Jinan, China; 5 Qilu Hospital of Shandong University, Jinan, China; 6 Jinan Central Hospital Affiliated to Shandong University, Jinan, China; 7 Provincial Key Lab of Mental Disorder, School of Medicine, Shandong University, China

## Abstract

This study was conducted to investigate the effects of oxytocin (OT) on visceral hypersensitivity/pain and mast cell degranulation and the underlying mechanisms. We found that oxytocin receptor (OTR) was expressed in colonic mast cells in humans and rats, as well as in human mast cell line-1 (HMC-1), rat basophilic leukemia cell line (RBL-2H3) and mouse mastocytoma cell line (P815). OT decreased 2,4,6-trinitrobenzene sulfonic acid (TNBS)-induced visceral hypersensitivity, colonic mast cell degranulation and histamine release after mast cell degranulation in rats. Also, OT attenuated the compound 48/80 (C48/80)-evoked histamine release in P815 cells and inward currents, responsible for the mast cell degranulation, in HMC-1, RBL-2H3 and P815 cells. Moreover, these protective effects of OT against visceral hypersensitivity and mast cell degranulation were eliminated by coadministration of OTR antagonist atosiban or a nonselective inhibitor of nitric oxide synthase (NOS), NG-Methyl-L-arginine acetate salt (L-NMMA). Notably, OT evoked a concentration-dependent increase of intracellular Ca^2+^ in HMC-1, RBL-2H3 and P815 cells, which was responsible for the activation of neuronal NOS (NOS1) and endothelial NOS (NOS3). Our findings strongly suggest that OT might exert the antinociception on colonic hypersensitivity through inhibition of mast cell degranulation via Ca^2+^-NOS pathway.

Oxytocin (OT), the neurohypophysial peptide well known for its role in parturition and lactation^1^, has been recognized to exert a wide spectrum of central and peripheral effects such as sexual and maternal behavior, human bonding and trust, and inflammation modulation^2^. It has been demonstrated that OT and OTR are expressed in bowel by our group^3^,^4^,^5^ and other studies^6^,^7^. Enteric OT, like that of brain, is restricted to neurons; however, enteric OTR is not exclusively neuronal. OT/OTR signaling is physiologically significant in the regulation of gastrointestinal motility and sensation, modulation of intestinal inflammation, regulation of the permeability of the mucosa to macromolecules, and maintenance of the mucosa^3^,^5^,^8^,^9^. Some studies have also demonstrated that OT plays an important role in visceral hypersensitivity/pain inhibition^10^,^11^. However, the mechanisms underlying the inhibitory effect of OT on visceral hypersensitivity/pain have not yet been fully elucidated.

Visceral hypersensitivity/abdominal pain is an essential symptom of irritable bowel syndrome (IBS)^12^, which correlates with the severity of the disease^13^. Both central nervous system mechanisms along the “brain-gut axis” and peripheral neuro-immune mechanisms constitute key concepts on pathophysiological mechanisms of abdominal pain in IBS. Mast cells, the sentinels of the immune system, may contribute to the pathogenesis of abdominal pain in IBS. The number of mast cells is increased in the colonic mucosa of IBS patients^14^. The severity and frequency of abdominal pain are correlated with the number of mast cells in close proximity to colonic nerves in IBS^15^. On the other hand, inflammation-induced visceral hypersensitivity is abolished in mast cell deficiency rats^16^. When activated, mast cells degranulate and release mediators that enhance the excitability of enteric and primary afferent neurons, leading to visceral hypersensitivity^17^. Histamine is a major inflammatory mediator released from mast cells when they degranulate, which could activate visceral afferents^17^ and enteric neurons^18^.

OT is widespread throughout the myenteric and submucous plexuses in the gastrointestinal tract. There is a closer proximity of mast cells to nerve fibers, and the number of mast cells per 10 fields <5 μm from nerves is 223% greater in IBS patients compared with healthy controls^15^,^17^. Therefore, we speculate that OT might suppress visceral hypersensitivity through inhibiting mast cell activation and degranulation.

Some evidence has suggested the participation of nitric oxide (NO) derived from NOS in the inhibition of mast cell activation/degranulation^19^ and histamine release^20^. NOS1 is expressed in 30% of human intestinal mast cells. NOS1, inducible NOS (NOS2) and NOS3 have been found in human mast cell (HMC)-1 cell line, NOS3 has been found in rat basophilic leukemia RBL-2H3 cell line and NOS2 is expressed in P815 mouse mastocytoma cell line. Furthermore, human intestinal mucosal mast cell (IMMC) express NOS1 and NOS3, while rat IMMC express only NOS3^21^. Furthermore, OT could elevate NOS activity in paraventricular nucleus^22^, dorsal root ganglion neurons^23^ and myenteric plexus^9^. We found that OT down-regulated visceral hypersensitivity in TNBS treated rats and inhibited mast cell degranulation. These preliminary data supported our hypothesis and provided new evidence that OT might inhibite mast cell activation and degranulation through activating NOS in mast cells.

## Results

### OTR was expressed in colonic mast cells in humans and rats

**Figure 1:**
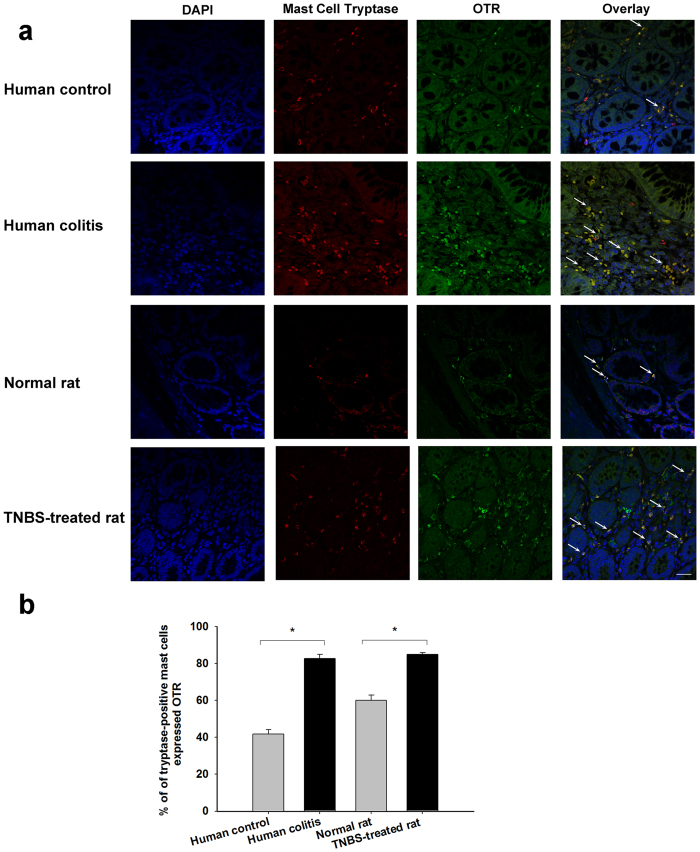
The OTR expression in colonic mast cells in humans and rats. (**a**) Confocal microscope images of three colon sections from a human control (a human normal colon section from a male patient with colon cancer), a male patient with ulcerative colitis, a normal rat and a TNBS-treated rat triple labeled for cell nucleus (blue), tryptase-positive mast cells (red) and the OTR (green). White arrows show examples of tryptase-positive mast cells in which the OTRs are expressed. Scale bar = 100 μm. (**b**) Summary data of tryptase-positive mast cells expressed OTR. *P < 0.05 versus control.

Immunofluorescence of human and rat colon tissues revealed that OT receptors were expressed in human and rat colonic mast cells (Fig. 1a). A total of 12 human normal colon sections from three male patients with colon cancer and 12 rat colon sections from three normal male rats were used in the study. A total of 100 and 120 mast cells in human and rat colon tissues were analyzed respectively. Confocal analysis of trinal immunofluorescence experiments revealed that 42.0% (42/100) and 62.6% (74/120) of tryptase-positive mast cells expressed OTR in human and rat normal tissues respectively (Fig. 1b). Besides, 8 human ulcerative colon sections from two male patients with ulcerative colitis were analyzed. The tryptase-positive mast cells significantly increased compared with that of human control group and the ratio of tryptase-positive mast cells expressed OTR reached 83.6% (92/110) (Fig. 1b). Furthermore, 8 colon sections from two TNBS-treated male rats were analyzed. The tryptase-positive mast cells significantly increased and the ratio of tryptase-positive mast cells expressed OTR reached 85.1% (97/114) (Fig. 1b).

### OTR was expressed in HMC-1, RBL-2H3 and P815 cells

**Figure 2:**
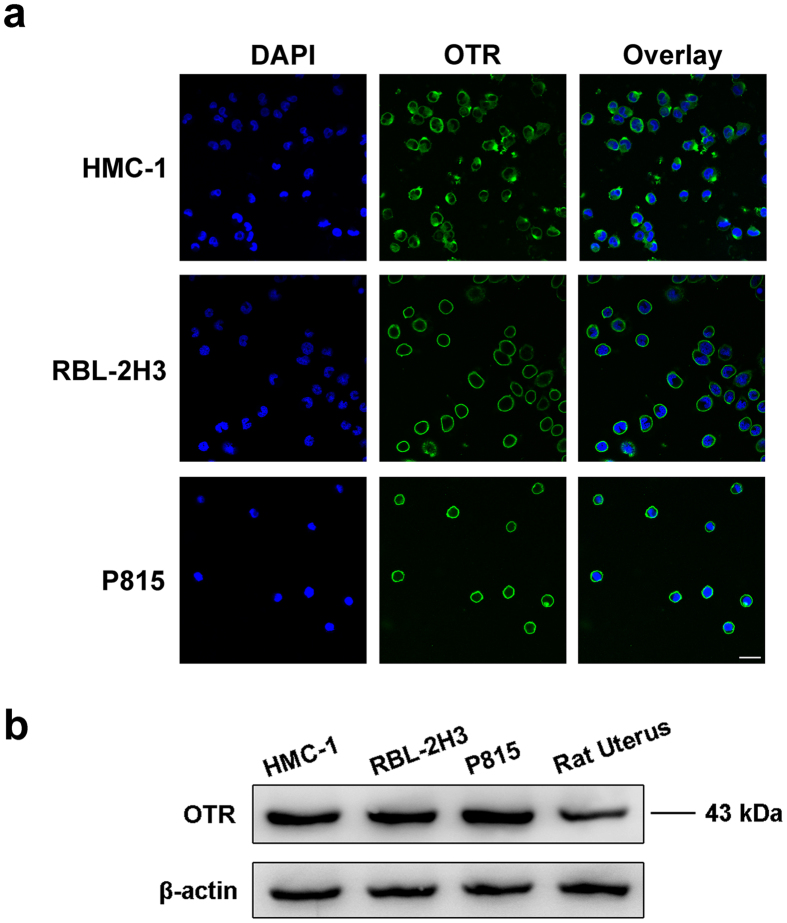
The OTR is expressed in HMC-1, RBL-2H3 and P815 cells. (**a**) Confocal microscope images of HMC-1, RBL-2H3 and P815 cells double labeled for cell nucleus (blue) and the OTR (green). Scale bar = 100 μm. (**b**) Detection of OTR protein in HMC-1, RBL-2H3 and P815 cells by Western blot. It is the representative immunoblots for OTR (43 kDa) and β-actin (43 kDa, loading control) in four groups, including HMC-1 cells, RBL-2H3 cells, P815 cells and rat uterus.

Next, we investigated if OTR was expressed in human, rat or mouse mast cell lines (HMC-1, RBL-2H3 or P815 cells). To do that, OTR was detected by immunofluorescence and Western blot. Immunofluorescence results demonstrated that all of the HMC-1, RBL-2H3 and P815 cells expressed OTR (Fig. 2a). Additionally, we found that the OTR protein was identified in HMC-1, RBL-2H3 and P815 cells, rat uterus as the positive control (Fig. 2b).

### OT alleviated TNBS-induced colonic hypersensitivity and colonic mast cell degranulation in rats

**Figure 3:**
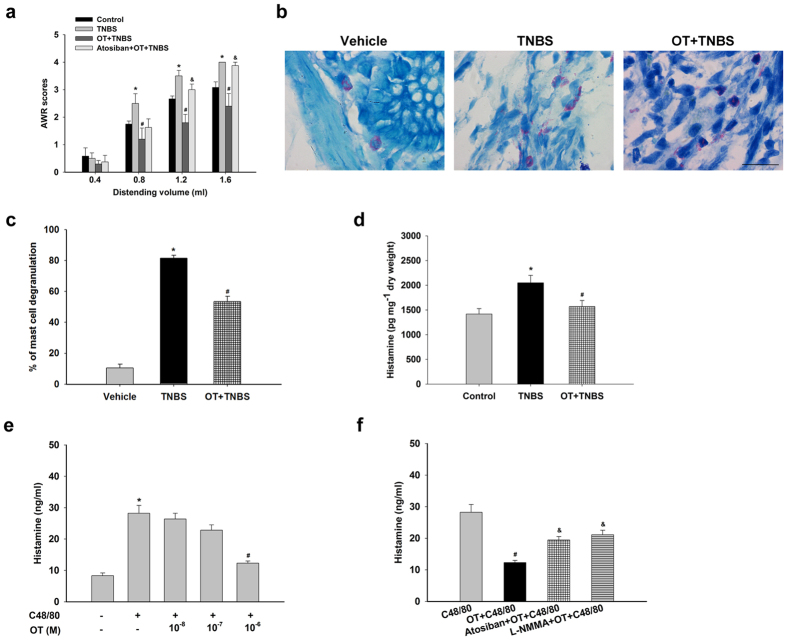
OT decreased abdominal withdrawal reflex scores to colorectal distention, mast cell degranulation and histamine release. (**a**) AWR scores of control and TNBS-treated rats to different colorectal distention volumes. AWR scores of TNBS-treated rats were remarkably greater than control group to colorectal distention with 0.8 ml, 1.2 ml and 1.6 ml water. AWR scores of OT-treated TNBS rats were obviously lower than TNBS group. Pretreatment with the OTR antagonist atosiban (0.5 mg/kg) reversed the AWR scores compared with OT + TNBS group. (**b**) Mast cells were identified in tissue sections by their characteristic granular, deep blue-purple metachromatic appearance against blue orthochromatic background tissue. Scale bar = 20 μm. (**c**) The rate of rat colonic mast cell degranulation in TNBS group (81.9%, 59/72) was markedly greater than control group (11.6%, 8/69). Intrarectal injection of OT (1 mg/kg/day) significantly reduced the rate of mast cell degranulation evoked by TNBS (30 mg) to 53.5% (38/71). (**d**) Spontaneous histamine release from colonic mucosa in saline, TNBS and OT + TNBS treated rats. TNBS led to a marked increment in basal histamine release of rat colon compared to normal saline group. OT treatment significantly inhibited the basal release of histamine compared to TNBS group. (**e**) C48/80 (10 μg/ml) caused a marked increment in histamine release compared to the control group. OT inhibited histamine release caused by C48/80 in a dose-dependent manner. (**f**) OTR antagonist atosiban (10^−6^ M) or nonselective NOS antagonist L-NMMA (10^−6^ M) reversed the inhibitory effect of oxytocin on C48/80-evoked histamine release. *P < 0.05 versus control, ^#^*P* < 0.05 versus TNBS or C48/80, ^&^*P* < 0.05 versus OT + TNBS or OT + C48/80.

IBS patients reported pain at lower volumes of balloon distension of the colon than normal subjects^24^. The injection of TNBS into the rat proximal colon resulted in a significant decrease in the sensory threshold of the non-inflamed distal colon to mechanical distention stimuli, which providing a useful tool for investigation of the pathophysiology and therapy of IBS^16^. In the study, an injection of TNBS into the rat proximal colon resulted in a significant increase in the sensitivity to colorectal distention on day 7 post-TNBS (*n* = 5) compared with vehicle treatment (*n* = 6) (Fig. 3a). Next, we investigated the effect of OT on colonic hypersensitivity. The results showed that intrarectal injection of OT (1 mg/kg/day) markedly decreased the TNBS-evoked sensitivity to colorectal distention on day 7 post-TNBS (*n* = 6) compared to the TNBS control rats (*n* = 5) (Fig. 3a). To further determine the role of OTR, the OTR antagonist atosiban (0.5 mg/kg) (*n* = 6), daily intrarectally injected to TNBS rats 30 min before OT (1 mg/kg) application, reversed the inhibitory effect of OT on TNBS-evoked visceral hypersensitivity (Fig. 3a).

Mast cells participated in the development of visceral hypersensitivity. When activated, mast cells degranulate and release mediators. In the study, mast cells were identified by metachromatic staining with toluidine blue. Mast cells were identified in tissue sections by their characteristic granular, deep blue-purple metachromatic appearance against blue orthochromatic background tissue. The results demonstrated that the rate of mast cell degranulation in TNBS group 81.9% (59/72) was significantly higher than that of vehicle group 11.6% (8/69) (Fig. 3b,c). More importantly, OT significantly reduced the rate of TNBS-evoked mast cell degranulation to 53.5% (38/71) (Fig. 3b,c).

### OT inhibited TNBS and C48/80 evoked histamine release in rat colon and P815 cells respectively

Mast cells played an important role in visceral hypersensitivity through releasing a variety of cytoines, chemokines, proteases, and histamine^17^. Histamine is a major biogenic amine released after the degranulation of mast cells^18^. To further determine the role of OT on mast cell degranulation. We examined the effects of OT on TNBS and C48/80 induced histamine release. The results showed that TNBS led to a marked increment in basal histamine release of rat colon compared to normal saline treatment. Histamine secretion from TNBS and saline group was 2050.2 ± 149.8 pg H/mg d.w. and 1417.6 ± 110.5 pg H/mg d.w. respectively (*P* < 0.05, TNBS group, *n* = 5; saline group, *n* = 6). OT treatment after TNBS-stimulated rat colon significantly inhibited the basal release of histamine (1568.3 ± 124.2 pg H/mg d.w.) compared to TNBS group (2050.2 ± 149.8 pg H/mg d.w.) (*P* < 0.05, OT+TNBS group, *n* = 6; TNBS group, *n* = 5) (Fig. 3d). In agreement with OT inhibition of colonic histamine release in rats, C48/80 (10 μg/ml) caused a marked increment in histamine release compared with that of the control group (28.2 ± 2.5 ng/ml, *n* = 7 in C48/80 group and 8.3 ± 0.9 ng/ml, *n* = 7 in control group, *P* < 0.05), which was attenuated by OT in a dose-dependent manner in P815 cells (Fig. 3e). To further confirm OTR involved in the inhibition of OT on histamine release, the OTR antagonist atosiban (10^−6^ M) was used. Unsurprisingly, it significantly blocked the inhibitory effect of OT on C48/80-evoked histamine release (Fig. 3f).

### OT inhibited C48/80-induced inward currents in HMC-1, RBL-2H3 and P815 cells

**Figure 4:**
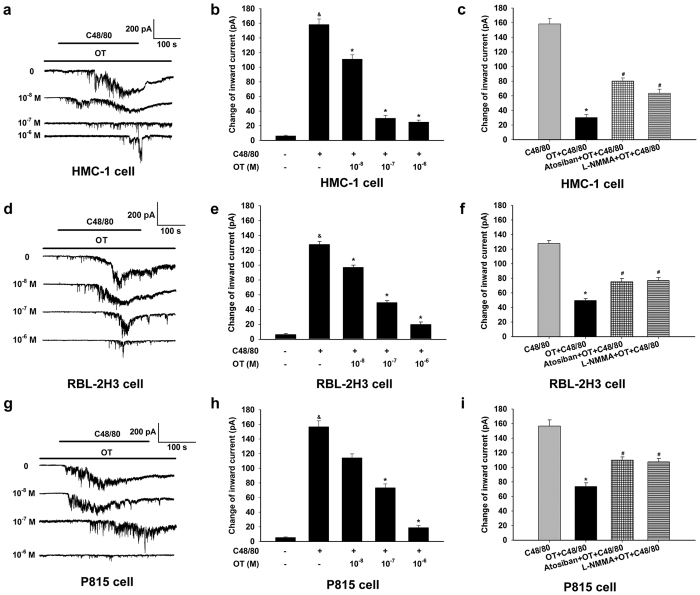
Reduction of OT on C48/80-evoked inward currents in HMC-1, RBL-2H3 and P815 cells. (**a**) Representative traces showing the inward currents with application of C48/80 (10 μg/ml) and OT (10^−8^ to 10^−6^ M) in HMC-1 cells. (**b**) Summary data of the change of inward currents following treatment with OT (10^−8^ to 10^−6^ M) in HMC-1 cells. 10^−8^, 10^−7^ and 10^−6^ M OT significantly inhibited the inward currents caused by C48/80. (**c**) OTR antagonist atosiban (10^−6^ M) or the NOS non-selective inhibitor L-NMMA (10^−6^ M) reversed the inhibitory effect of OT on the 10 μg/ml C48/80-induced inward currents in HMC-1 cells. (**d**) Representative traces showing the inward currents with application of C48/80 and OT (10^−8^ to 10^−6^ M) in RBL-2H3 cells. (**e**) Summary data of the change of inward currents following treatment with OT (10^−8^ to 10^−6^ M) in RBL-2H3 cells. 10^−8^, 10^−7^ and 10^−6^ M OT significantly inhibited the inward currents caused by C48/80. (**f**) OTR antagonist atosiban (10^−6^ M) or the NOS non-selective inhibitor L-NMMA (10^−6^ M) reversed the inhibitory effect of OT on the C48/80-induced inward currents in RBL-2H3 cells. (**g**) Representative traces showing the inward currents with application of C48/80 (10 μg/ml) and OT (10^−8^ to 10^−6^ M) in P815 cells. (**h**) Summary data of the change of inward currents following treatment with OT (10^−8^ to 10^−6^ M) in P815 cells. 10^−7^ and 10^−6^ M OT significantly inhibited the inward currents caused by C48/80. (**i**) OTR antagonist atosiban (10^−6^ M) or the NOS non-selective inhibitor L-NMMA (10^−6^ M) reversed the inhibitory effect of OT on the C48/80-induced inward currents in P815 cells. ^&^*P* < 0.05 versus baseline, *P < 0.05 versus C48/80, ^#^*P* < 0.05 versus OT + C48/80.

C48/80 activates the Ca^2+^-permeable channel in mast cells, which leads to mast cell degranulation. To investigate whether OT attenuated the inward currents caused by C48/80, patch-clamp recordings were performed. The HMC-1 cells were initially held at a −60 mV command potential. Inward currents appeared within 10 s after the addition of C48/80 (10 μg/ml) and increased to reach a peak at about 4 min, after which the currents declined (Fig. 4a). Five minute following C48/80 alone, the average value during 2–6 min of inward currents was markedly increased compared with that of the baseline group, which was attenuated by OT pre-treated before C48/80 in a dose-dependent manner in HMC-1 cells (Fig. 4b). The RBL-2H3 and P815 cells were also initially held at a −60 mV command potential. Five minute following C48/80 alone, the average value during 2–6 min of inward currents was markedly increased compared with that of the baseline group, which was largely attenuated by OT pre-treated before C48/80 in a dose-dependent manner in RBL-2H3 and P815 cells (Fig. 4d,e,g,h).

### Role of NOS in the inhibition of OT on mast cell degranulation

Both endogenous and exogenous NO could inhibit mast cell degranulation. Besides, OT could activate the NOS1 and NOS3. To determine if NOS was involved in the inhibition of OT on mast cell degranulation, we applied the NOS non-selective inhibitor L-NMMA (10^−6^ M) to HMC-1, RBL-2H3 and P815 cells. In P815 cells, pretreatment with non-specific NOS inhibitor L-NMMA (10^−6^ M) markedly reversed the inhibitory effect of OT (10^−6^ M) on C48/80-evoked histamine release from 12.3 ± 0.7 ng/ml (OT + C48/80 group, *n* = 7) to 21.12 ± 1.43 ng/ml (L-NMMA + OT + C48/80 group, *n* = 7) (*P* < 0.05) (Fig. 3f). Furthmore, L-NMMA (10^−6^ M) significantly attenuated the inhibitory effects of OT on C48/80-induced inward currents in HMC-1, RBL-2H3 and P815 cells (Fig. 4c,f,i).

### OT evoked an increase in intracellular Ca^2+^ in HMC-1, RBL-2H3 and P815 cells

**Figure 5:**
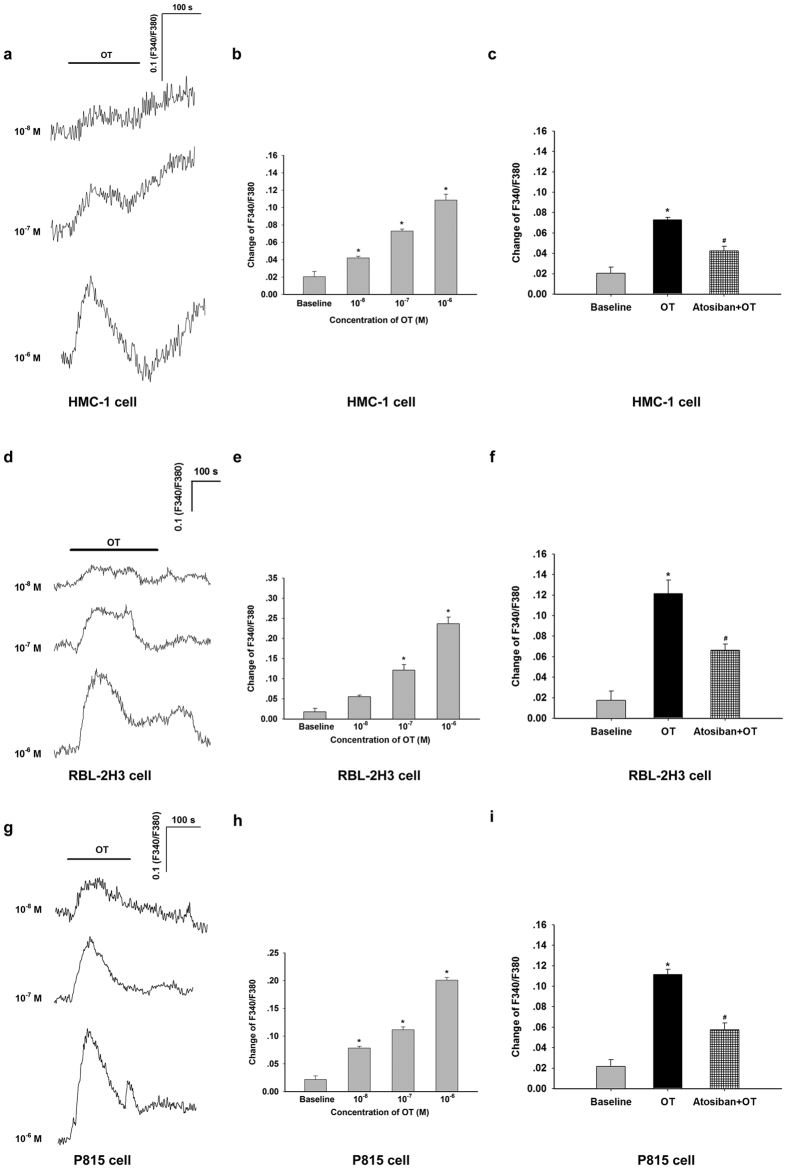
Increase of OT on intracellular Ca^2+^ level in HMC-1, RBL-2H3 and P815 cells. (**a**) The time course and dose-dependence of the effect of OT (10^−8^ to 10^−6^ M) on the intracellular Ca^2+^ level in HMC-1 cells. (**b**) Summary data showing the effects of different concentrations of OT (10^−8^ to 10^−6^ M) on the change of fluorescence ratio in HMC-1 cells. (**c**) OTR antagonist atosiban (10^−6^ M) decreased OT-evoked the increase of intracellular Ca^2+^ in HMC-1 cells. (**d**) The time course and dose-dependence of the effect of OT (10^−8^ to 10^−6^ M) on the intracellular Ca^2+^ level in RBL-2H3 cells. (**e**) Summary data showing the effects of different concentrations of OT (10^−8^ to 10^−6^ M) on the change of fluorescence ratio in RBL-2H3 cells. (**f**) OTR antagonist atosiban (10^−6^ M) decreased OT-evoked the increase of intracellular Ca^2+^ in RBL-2H3 cells. (**g**) The time course and dose-dependence of the effect of OT (10^−8^ to 10^−6^ M) on the intracellular Ca^2+^ level in P815 cells. (**h**) Summary data showing the effects of different concentrations of OT (10^−8^ to 10^−6^ M) on the change of fluorescence ratio in P815 cells. (**i**) OTR antagonist atosiban (10^−6^ M) decreased OT-evoked the increase of intracellular Ca^2+^ in P815 cells. *P < 0.05 versus baseline, ^#^*P* < 0.05 versus OT.

NOS1 and NOS3 were stimulated by intracellular Ca^2+^. To further investigate the underlying mechanism of the inhibition of OT on mast cell degranulation, intracellular Ca^2+^ was detected by calcium imaging. Effects of OT on the fura-2 fluorescence ratio (F340/F380), due to changes in intracellular Ca^2+^ concentration, were tested over a dose range of 10^−8^ M −10^−6^ M. The results demonstrated that OT caused a dose-dependent increase in intracellular Ca^2+^ in HMC-1, RBL-2H3 and P815 cells. In HMC-1 cells, the intracellular Ca^2+^ reached the highest level at about 60 s after OT administration, after which the level of intracellular Ca^2+^ declined (Fig. 5a). After application of OT (10^−8^, 10^−7^, and 10^−6^ M), the F340/F380 ratio increased in a dose-dependent manner in HMC-1 cells (Fig. 5b). In RBL-2H3 and P815 cells, the intracellular Ca^2+^ also increased in a dose-dependent manner after application of OT (10^−8^, 10^−7^, and 10^−6^ M) and reached the highest level at about 60 s after OT administration (Fig. 5d,e,g,h). To further confirm OTR participated in OT-evoked the increase of intracellular Ca^2+^, the OTR antagonist atosiban (10^−6^ M) was used. Unsurprisingly, it largely blocked the OT-evoked the increase of intracellular Ca^2+^ in HMC-1, RBL-2H3 and P815 cells (Fig. 5c,f,i).

## Discussion

This study demonstrated that OTR was expressed in colonic mast cells in both humans and rats and it was contained in HMC-1, RBL-2H3 and P815 cells. Importantly, we found that exogenous OT decreased the TNBS-induced visceral hypersensitivity and mast cell degranulation in rats and it attenuated the C48/80-evoked histamine release and inward currents in HMC-1, RBL-2H3 and P815 cells as well. These effects were blocked by OTR antagonist atosiban or the nonselective NOS inhibitor L-NMMA. Furthermore, we found that OT could increase intracellular Ca^2+^ production in HMC-1, RBL-2H3 and P815 cells.

The encoded OTR belongs to the class I G protein-coupled receptor family. In the peripheral system, OTR is present in the mammary gland, and in both the myometrium and endometrium of the uterus. OTR has also been identified in other tissues, including the kidney, heart, adipocytes, pancreas, and thymus^1^. Additionally, OTR is expressed by the majority of myenteric neurons, submucosal neurons, mucosal epithelium cells, and submucosal venules in the bowel in rats^6^. In this study, we confirmed the expression of OTR in the colonic mast cells in humans and rats using immunofluorescence. Moreover, the expression of OTR was demonstrated in HMC-1, RBL-2H3 and P815 cells using immunofluorescence and Western blot.

Plasma OT concentrations range from 1.70 pmol/L to 45.0 pmol/L in humans^25^. It is elevated by several times during the second stage of labor^26^, breast stimulation of lactating women^27^, sexual arousal in both women and men^28^, or headache in patients^29^. A preclinical toxicity screen indicates that intrathecal administration of OT causes a 500-fold increase in plasma OT levels from a baseline of approximately 100 pmol/L to 50000 pmol/L without neurotoxicity in dogs^30^. If plasma OT concentrations are increased by 500 times in rats, it will reach to the working concentration (10^−7^ M) of OT in this study. It is also important to note that OT is widespread throughout the myenteric and submucous plexuses in the human and guinea pig gastrointestinal tract^7^,^31^, and there is a much closer proximity of nerve fibers to mast cells^15^,^17^. Besides, membrane to membrane contacts between mast cells and nerve fibers are occasionally observed, and activated mast cells with degranulation polarized toward the nerves are often found in the close proximity (0–10 μm) of nerve trunks in IBS specimens^17^. These mean that the OT concentration in the microenviroment of colonic mucosal mast cells might be higher than that in plasma. Thus, it can explain why physiological concentration of OT in plasma, ranging from 10^−12^ − 10^−8^ M^32^, failed to exert significant effects on the mast cells in some situations in this study.

Exogenous OT applied peripherally attenuates visceral hypersensitivity/pain in human samples and animal models. Intravenous infusion of OT significantly increases thresholds of colonic visceral perception in patients with IBS^10^. Intraperitoneal administration of OT reduces the visceral hypersensitivity in rats^33^. OT and OT analogues display important analgesic effects on formalin-induced tonic continuous pain response and intra-colonic TNBS evoked chronic visceral hypersensitivity respectively in mice^34^. Consistent with the findings above, in this work, we demonstrated that OT could decrease colonic visceral hypersensitivity caused by TNBS in rats.

Mast cell activation is involved in visceral hypersensitivity, one of the main characteristics of the IBS^35^. Mucosal mast cell counts correlate with visceral hypersensitivity in IBS^15^. Furthermore, mast cell activation can promote visceral hypersensitivity in functional gastrointestinal disorders and inflammatory bowel disease^36^. On the other hand, the mast cell stabilizer could decrease visceral hypersensitivity in patients with IBS^35^ and TNBS-induced visceral hypersensitivity in rats^37^. In addition, TNBS failes to elicit visceral hypersensitivity in mast cell deficiency rats^16^.

In response to various stimuli, mast cells degranulate, the functional status of mast cells^38^, and release histamine, tryptase, cytokines, growth factors, chemokines and lipid mediators^39^. Mediators released from colonic mucosal biopsies of IBS patients can activate human submucosal neurons^18^ and rat nociceptive visceral sensory nerves^17^. Moreover, these mediators may also activate visceral afferent neurons in rats and mice and cause visceral hypersensitivity in mice^40^. In fact, these mediators induced effects are mainly mediated by proteases and histamine^18^,^40^. Histamine mainly exists in mast cells, and its release is considered as a reliable indicator of mast cell degranulation^41^. Besides, histamine is reported to cause activation of submucosal neurons mainly mediated by histamine H_1_ receptors on the neurons in rat^42^. Histamine has been demonstrated to increases Nav1.8 expression in primary afferent neurons via histamine H_2_ receptor-mediated pathway and thereby contributes to neuropathic pain^43^.

The mechanisms of mast cell activation can be categorized as immunoglobulin E (IgE)-dependent and IgE-independent^21^. The common pathway is through the crosslinking of specific IgE bound to FcεRI receptors. The second pathway, mast cells can be activated with nonimmunogenic stimuli such as spermine, neuropeptides substance P, and C48/80^44^. It has been identified that intraperitoneal injection of C48/80 to the rat causes mast cell degranulation associated with histamine release in the mesentery^45^. Furthermore, it has been reported that P815 cells and RBL-2H3 cells are induced to degranulate in response to compound 48/80, releasing histamine and β-hexosaminidase respectively^46^.

Both IgE-dependent and IgE-independent activation of both human and rodent mast cells are characterized by an influx of extracellular Ca^2+^ that is essential for the release of both preformed (granule-derived) mediators and newly generated autacoids and cytokines^47^. Antigenic stimulation of mast cells is best understood via the crosslinking of specific IgE bound to FcεRI receptors. FcεRI aggregation can induce the activation of phosphoinositide-specific phospholipase C (PLC). PLC breaks downs phosphatidylinositol-4,5-bisphosphate (PIP2) to generate two second messengers, inositol-1,4,5-triphosphate (IP_3_) and DAG. IP_3_ binds to IP_3_ receptors located on the surface of the endoplasmic reticulum (ER) and activates the release of Ca^2+^ 48. The mechanism, known as Ca^2+^-induced Ca^2+^ release, can lead to prolonged propagation of Ca^2+^ signals. The major pathway of Ca^2+^ influx is through calcium release activated Ca^2+^ (CRAC) channels, also known as store operated channels (SOC), in mast cells^47^. C48/80 is widely used in animal and tissue models as a non-IgE dependent mast cell activator. C48/80 bypasses the PLC, acts as receptor mimetic agents, and induces mast cell degranulation by directly activating the GTP-binding proteins (Gi proteins) in a receptor-independent manner^49^. C48/80 activates the Ca^2+^-permeable channel in rat peritoneal mast cells, and the channel is not activated by depolarization but by second messengers. C48/80-induced whole cell current is a mixture of currents through, at least, the Ca^2+^-permeable channel and the cation-selective channel^50^. Although the inflammatory changes and the clinical manifestations are similar for both pathways, there are some differences in the mast cell activation between immunological and nonimmunological stimuli. With C48/80 treatment, histamine release is faster than IgE induction and independent of external Ca^2+^ 51. In addition, high concentration of C48/80 can induce almost a 90% release of histamine from mast cells, while IgE induced histamine release rarely reaching 50%^52^.

In the study, mast cell degranulation is characterized by the rate of TNBS-evoked mast cell degranulation, TNBS or C48/80 induced histamine release, and C48/80-evoked inward currents, and we found that OT inhibited mast cell degranulation in these aspects. Evidence indicates that OT may reduce both granulated and degranulated mast cells against ischemia/reperfusion (I/R) injury in urinary bladder tissue in rats^53^. G. Csaba showed a inhibition of OT on the level of adrenocorticotrophine (ACTH), important in chemotaxis and phagocytosis, in intraperitoneal mast cells in rats^54^. In line with these previous findings, we demonstrated the inhibitiory effect of OT on mast cell degranulation.

NO is mainly synthesized from L-arginine by NOS. There are three NOS isoforms, NOS1, NOS2, and NOS3. NOS1 and NOS3, the Ca^2+^-dependent members, are expressed in HMC-1 cells. NOS3 is expressed in RBL-2H3 cells^55^. Mast cell functions could be regulated by nitric oxide. Both endogenous and exogenous NO inhibit mast cell degranulation. NOS inhibitor, L-NAME, increases rat mast cell protease II activity^56^ and also elicites mast cell degranulation^57^. Pretreatment of enriched rat peritoneal mast cells with the NOS inhibitor, L-NMMA, markedly enhances *E. coli* LPS-evoked histamine release^57^. On the other hand, the NO donor, sodium nitroprusside (SNP), inhibites release of histamine evoked by C48/80^58^. NO donors such as sodium nitroprusside, spermine-NO, and SIN1 significantly reduce mast cell degranulation in the mesentery after ischemia/reperfusion in rats^59^.

OT increases the activity of NOS in medial basal hypothalamus^60^. By acting on its GPCRs coupled to Gq, OT activates PLC, which leads to an increase in intracellular Ca^2+^. Ca^2+^ binds to calmodulin, and the Ca^2+^/calmodulin complex (CaM) stimulates NOS3, leading to NO production in human endothelial cells^61^. Consistent with the findings above, in this study, pre-treatment with the non-selective inhibitor L-NMMA markedly reversed the inhibitory effect of OT on C48/80-evoked histamine release in P815 cells and C48/80-induced inward currents in HMC-1, RBL-2H3 and P815 cells. For the first time, we found that OT increased the production of Ca^2+^ and produced inhibition of mast cell degranulation in a NOS-dependent manner in the mast cells. So it is possible that OT exerted the inhibition of mast cell degranulation by Ca^2+^ -NOS pathway in mast cells.

**Figure 6:**
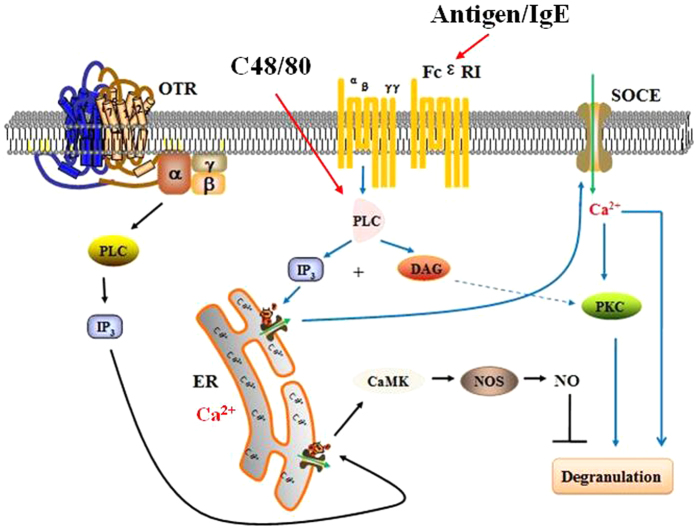
Proposed mechanisms for the inhibition of OT on mast cell degranulation. OT activates PLC, induces the release of Ca^2+^ from endoplasmic reticulum, then triggers NOS producing NO and thus inhibites mast cell degranulation.

Our results indicate that exogenous OT could attenuate TNBS-induced visceral hypersensitivity and mast cell degranulation in rats, and the inhibition of OT on mast cell degranulation was mediated by activation of the Ca^2+^-NOS pathway (Fig. 6). These findings provide new insights into the possible peripheral mechanism of visceral analgesic action of OT which has potential therapeutic value as a pain modulator and antinociceptive agent.

## Material and Methods

### Ethics statement

In the present study, human colon tissues were obtained from three male patients with colon cancer and two male patients with ulcerative colitis. All studies using this human material were in accordance with the Declaration of Helsinki and approved by the local Ethics committee of Shandong University, China. Informed consent was obtained from all participants before each colon tissue donation.

### Animals

Wistar male rats (200–220 g) were provided by the Experimental Animal Center of Shandong University. All experimental procedures were conducted in accordance with the Guidelines for the Care and Use of Laboratory Animals of Shandong University, and the study was approved by the Medical Ethics Committee for Experimental Animals, Shandong University, China

### TNBS-induced hypersensitivity and behavioral response

After 16–18 h fasting, the animals were anesthetized with an intraperitoneal injection of pentobarbital sodium (50–90 mg/kg). Using a polyethylene tubing, 30 mg (per rat) of TNBS in 25% ethanol (total volume, 0.8 ml) was instilled into the colon 8 cm proximal to the anus. An equivalent volume of ethanol was administered into control rats. Rats were kept in a vertical position for several minutes to avoid leakage of the instilled intracolonic solutions. A total of 72 Wistar male rats treated with TNBS were randomly divided into 3 groups: a model control group, an OT treatment group and an atosiban + OT group (*n* = 24 per group). The rats daily received intrarectal treatment with 0.9% NaCl, OT and atosiban + OT respectively for 7 days. And then visceral pain testing was performed. A 5-cm latex balloon was inserted through the anus and placed in the distal colon at 5 cm from the anus. After 30-min acclimation, graded colorectal distention (CRD) administrations (0.4, 0.8, 1.2, and 1.6 ml) of 20-sec durations were performed every 5 min for two times. The visceral pain in response to CRD was assessed by measuring the abdominal withdrawal reflex (AWR) by a blinded observer and AWR was scored either 0 (normal behavior), 1 (slight head movement), 2 (contraction of abdominal muscles), 3 (lifting of abdominal wall) or 4 (body arching and lifting of pelvic structures).

### Cell culture

HMC-1 (human mast cell line-1), RBL-2H3 (rat basophilic leukemia cell line) and P815 (mouse mastocytoma cell line) cells were grown in Iscove’s modified Dulbecco’s medium (IMDM; Gibco), minimum essential medium (MEM; Gibco) and RPMI 1640 medium (Gibco) respectively, supplemented with 100 U/ml of penicillin (Gibco), 100 g/ml of streptomycin (Gibco), and 10% fetal bovine serum (Gibco) at 37 °C in 5% CO_2_ with 95% humidity.

### Immunofluorescence

HMC-1, RBL-2H3 or P815 cells adhered to the bottom of round glass panes and were rinsed three times with phosphate-buffered saline (PBS), soaked in 4% paraformaldehyde for 30 min at 22–25 °C, and incubated with 10% donkey serum for 1 h. Then the cells were incubated with the primary antibody, a goat anti-OTR antibody (1 : 50, ab87312; abcam), for 16–18 h at 4 °C. The cells were washed three times with PBS and incubated with a secondary antibody, Alexa Fluor 488-labeled donkey anti-goat IgG antibody (1 : 500, A11055; Invitrogen), for 1 h at 22–25 °C. After washing three times with PBS, the cells were incubated with DAPI (1: 2000). After washed with PBS three times, the immunopositive cells were detected using confocal microscopy (LSM780; Carl Zeiss).

Four-micron-thick sections were prepared from 4% paraformaldehyde fixed, paraffin-embedded colonic segments that were removed from the distal colon of male humans and rats. Sections were de-waxed and hydrated. After antigen retrieval, sections were incubated with 10% donkey serum for 1 h. Then the sections were incubated with a primary antibody mixture including a goat anti-OTR antibody (1 : 50, ab87312; abcam) and a rabbit anti-mast cell tryptase antibody (1 : 500, ab134932; abcam) for 16–18 h at 4 °C. The sections were washed three times with PBS and incubated with a secondary antibody mixture composed of Alexa Fluor 488-labeled donkey anti-goat IgG antibody (1 : 500, A11055; Invitrogen) and Alexa Fluor 568-labeled donkey anti-rabbit IgG antibody (1 : 500, A10042; Invitrogen) for 1 h at 22–25 °C. After washing three times with PBS, the sections were incubated with DAPI (1: 2000). After washed with PBS three times, the immunopositive cells on the sections were detected using confocal microscopy (LSM780; Carl Zeiss).

### Western blot

Total protein concentration in the supernatant was determined with Bicinchoninic Acid assay (Beyotime biotechnology, China). The supernatant was electrophoresed and transferred to nitrocellulose membrane. The membrane was incubated in blocking buffer [5% non-fat dry milk in tween/tris-buffered salt solution (TTBS)] for 1 h at 20 °C, washed in TTBS, and incubated overnight with rabbit anti-OTR antibody (1 : 1000, ab181077; abcam). After multiple washes, the membranes were incubated at 20 °C for 1 h with donkey anti-rabbit IgG secondary antibody (1: 2000, A0208; Beyotime, Nantong, China) conjugated with HRP. The immunopositive proteins on the membrane were detected by ECL plus (Millipore, Bedford, USA).

### Toluidine blue staining

To assist in differentiating mast cells from other inflammatory cells, toluidine blue staining was used in this study^62^,^63^. Serialized 4-μm-thick sections were deparaffinized, rehydrated, and stained with 0.5% toluidine blue (D034; NanJing Jiancheng Bioengineering Institute, Nanjing, China).

### Histamine assay

P815 cells were pretreated with various concentrations of OT for 10 min before C48/80 stimulation. In some experiments, P815 cells were preincubated with OTR or NOS blocker for 10 min before OT (10^−7^ M) treatment. The cells were separated from the released histamine by centrifugation at 2000 rpm for 10 min at 4 °C. The levels of histamine in the supernatant were measured by a commercial ELISA kit (H171; NanJing Jiancheng Bioengineering Institute, Nanjing, China).

To test the concentration of histamine spontaneously released from rat colon, about 0.1 g colon tissue was taken from TNBS or OT + TNBS rats and incubated in 2 ml Hank’s fluid. After a pre-incubation period of 25 min, 200 μl of the incubation medium were removed to detect basal release of histamine within this time period. Then, the immediate protein denaturation of the supernatants was performed by heating the samples at 95 °C for 7 min. After heat inactivation, all samples were centrifuged at 200 g for 10 min, and 150 μl was transferred to plastic tubes for storage at −20 °C. The tissue particles were frozen, lyophilized for 24 h, and weighed on a microbalance. Histamine released was detected by using a highly sensitive and specific radioimmunoassay.

### Whole-cell patch clamp recording

Whole-cell patch-clamp recordings were performed using an Axon Instruments Multiclamp 700B amplifier (Molecular Devices, New York, NY, USA) interfaced to Digidata 1440A with the pClamp 10.2 software (Molecular Devices). Glass pipettes filled with an intracellular saline had a resistance of 5–7 MΩ. The external solution was Krebs saline. All recordings were conducted at 30 °C.

### Calcium Imaging

The HMC-1, RBL-2H3 and P815 cells were adhered to the bottom of round glass panes, incubated with the calcium-sensitive dye fura-2/AM ester (1 μM, 1 mM stock in dimethylsulphoxide (DMSO)) for 60 min in imaging bath solution, and then washed three to four times for 30 min with Krebs saline. The HMC-1, RBL-2H3 and P815 cells were exposed to vehicle or different concentrations of OT (10^−8^–10^−6^ M). All imaging experiments were performed in the dark, at room temperature (20–25 °C). Fura-2 fluorescence was recorded at 510 nm during alternating excitation at 340 and 380 nm at 1 Hz using a monochromater (Polychrome V, FEI Company, Hillsboro, Oregon, USA). Regions of interest were defined on a computer connected to a CCD camera, and ratio of emission at 510 nm from excitation at 340 and 380 was analyzed. Only cells with a resting 340/380 fluorescence ratio of 0.55 to 0.90 were included.

### Solutions

Krebs saline contained the following reagents (in mM): 118.1 NaCl, 4.8 KCl, 25.0 NaHCO_3_, 1.0 NaH_2_PO_4_, 1.2 MgSO_4_, 11.1 glucose, and 2.5 CaCl_2_. The intracellular saline was composed of the following reagents (in mM): 110–115.00 KMeSO_4_, 9.00 NaCl, 0.09 CaCl_2_, 1.00 MgCl_2_, 10.00 HEPES, 0.20 Na_3_GTP, and 0.20 BAPTA.K_4_, with KOH to bring the pH to 7.3.

### Chemicals

Atosiban was purchased from Ferring AB (Malmoe, Sweden). Fura-2AM and DAF-FM diacetate were purchased from Life Technologies (Eugene, Oregon, USA). C48/80, OT, TNBS and L-NMMA were purchased from Sigma-Aldrich Corp (St Louis, MO, USA). Fura-2 and DAF-FM diacetate were dissolved in DMSO to make the stock solution, with final DMSO concentration 0.1%. Atosiban, C48/80, OT, TNBS and L-NMMA were dissolved in normal saline to make the stock solution. All stock solutions were finally diluted into extracellular saline to make the final working concentrations.

### Statistical analysis

For patch clamp recording, the change of inward current following treatment with Krebs saline was taken as the baseline. The effects of OT were measured in paired experiments where total outward currents were measured before and after application of the hormone. For calcium imaging experiments, differences between the change of intracellular Ca^2+^ following treatment with Krebs saline (baseline group) and different doses of OT were compared.

All the data were given as mean ± SEM, and *n* indicates the number of rats in visceral hyperalgesia detection experiments or cells in other experiments. One-way analysis of variance (ANOVA) or *t*-tests were used to test for differences between groups. When a statistically significant (*P* ≤ 0.05) treatment was identified, post hoc tests were used to establish where the differences lie. For all analyses, *P* < 0.05 was accepted as evidence of significance.

## Additional Information

**How to cite this article**: Gong, L. *et al*. The antinociception of oxytocin on colonic hypersensitivity in rats was mediated by inhibition of mast cell degranulation via Ca^2+^-NOS pathway. *Sci. Rep.* **6**, 31452; doi: 10.1038/srep31452 (2016).

## References

[b1] Gimpl G. & Fahrenholz F. The oxytocin receptor system: structure, function, and regulation. Physiol Rev 81, 629–683 (2001). 11274341 10.1152/physrev.2001.81.2.629

[b2] Lee H. J., Macbeth A. H., Pagani J. H. & Young W. S. 3rd. Oxytocin: the great facilitator of life. Prog Neurobiol 88, 127–151 (2009). 19482229 10.1016/j.pneurobio.2009.04.001 PMC2689929

[b3] Che T. . Oxytocin hyperpolarizes cultured duodenum myenteric intrinsic primary afferent neurons by opening BK(Ca) channels through IP(3) pathway. J Neurochem 121, 516–525 (2012). 22356163 10.1111/j.1471-4159.2012.07702.x

[b4] Feng M. . Estradiol upregulates the expression of oxytocin receptor in colon in rats. Am J Physiol Endocrinol Metab 296, E1059–E1066 (2009). 19258489 10.1152/ajpendo.90609.2008

[b5] Chen D. . Oxytocin evokes a pulsatile PGE2 release from ileum mucosa and is required for repair of intestinal epithelium after injury. Sci Rep 5, 11731 (2015). 26159321 10.1038/srep11731 PMC4498177

[b6] Welch M. G., Tamir H., Gross K. J., Chen J., Anwar M. & Gershon M. D. Expression and developmental regulation of oxytocin (OT) and oxytocin receptors (OTR) in the enteric nervous system (ENS) and intestinal epithelium. J Comp Neurol 512, 256–270 (2009). 19003903 10.1002/cne.21872 PMC3097117

[b7] Yu Q. . Oxytocin is expressed by both intrinsic sensory and secretomotor neurons in the enteric nervous system of guinea pig. Cell Tissue Res 344, 227–237 (2011). 21437658 10.1007/s00441-011-1155-0

[b8] Welch M. G., Margolis K. G., Li Z. & Gershon M. D. Oxytocin regulates gastrointestinal motility, inflammation, macromolecular permeability, and mucosal maintenance in mice. Am J Physiol Gastrointest Liver Physiol 307, G848–G862 (2014). 25147234 10.1152/ajpgi.00176.2014 PMC4200316

[b9] Li J. . Oxytocin down-regulates mesenteric afferent sensitivity via the enteric OTR/nNOS/NO/KATP pathway in rat. Neurogastroenterol Motil 27, 51–62 (2015). 25346204 10.1111/nmo.12469

[b10] Louvel D. . Oxytocin increases thresholds of colonic visceral perception in patients with irritable bowel syndrome. Gut 39, 741–747 (1996). 9014776 10.1136/gut.39.5.741 PMC1383401

[b11] Ray K. Pain Oxytocin analogues have potential in relieving chronic abdominal pain. Nat Rev Gastroenterol Hepatol 11, 202 (2014). 10.1038/nrgastro.2014.20 24535327

[b12] Thompson W. G., Longstreth G. F., Drossman D. A., Heaton K. W., Irvine E. J. & Muller-Lissner S. A. Functional bowel disorders and functional abdominal pain. Gut 45 Suppl 2, II43–II47 (1999). 10457044 10.1136/gut.45.2008.ii43 PMC1766683

[b13] Sandler R. S., Drossman D. A., Nathan H. P. & McKee D. C. Symptom complaints and health care seeking behavior in subjects with bowel dysfunction. Gastroenterology 87, 314–318 (1984). 6735075

[b14] Chadwick V. S. . Activation of the mucosal immune system in irritable bowel syndrome. Gastroenterology 122, 1778–1783 (2002). 12055584 10.1053/gast.2002.33579

[b15] Barbara G. . Activated mast cells in proximity to colonic nerves correlate with abdominal pain in irritable bowel syndrome. Gastroenterology 126, 693–702 (2004). 14988823 10.1053/j.gastro.2003.11.055

[b16] Ohashi K., Sato Y., Kawai M. & Kurebayashi Y. Abolishment of TNBS-induced visceral hypersensitivity in mast cell deficient rats. Life Sci 82, 419–423 (2008). 18222490 10.1016/j.lfs.2007.11.027

[b17] Barbara G. . Mast cell-dependent excitation of visceral-nociceptive sensory neurons in irritable bowel syndrome. Gastroenterology 132, 26–37 (2007). 17241857 10.1053/j.gastro.2006.11.039

[b18] Buhner S. . Activation of human enteric neurons by supernatants of colonic biopsy specimens from patients with irritable bowel syndrome. Gastroenterology 137, 1425–1434 (2009). 19596012 10.1053/j.gastro.2009.07.005

[b19] Chau J. Y. . Malaria-associated L-arginine deficiency induces mast cell-associated disruption to intestinal barrier defenses against nontyphoidal Salmonella bacteremia. Infect Immun 81, 3515–3526 (2013). 23690397 10.1128/IAI.00380-13 PMC3811760

[b20] Parikh V. & Singh M. Possible role of nitric oxide and mast cells in endotoxin-induced cardioprotection. Pharmacol Res 43, 39–45 (2001). 11207064 10.1006/phrs.2000.0750

[b21] Sekar Y., Moon T. C., Munoz S. & Befus A. D. Role of nitric oxide in mast cells: controversies, current knowledge, and future applications. Immunol Res 33, 223–239 (2005). 16462000 10.1385/IR:33:3:223

[b22] Melis M. R., Succu S., Iannucci U. & Argiolas A. Oxytocin increases nitric oxide production in the paraventricular nucleus of the hypothalamus of male rats: correlation with penile erection and yawning. Regul Pept 69, 105–111 (1997). 9178353 10.1016/s0167-0115(97)00002-5

[b23] Gong L. . Oxytocin-induced membrane hyperpolarization in pain-sensitive dorsal root ganglia neurons mediated by Ca(2+)/nNOS/NO/KATP pathway. Neuroscience 289, 417–428 (2015). 25617653 10.1016/j.neuroscience.2014.12.058

[b24] Gebhart G. F. Visceral pain-peripheral sensitisation. Gut 47 Suppl 4, iv54–iv55; discussion iv58 (2000). 11076915 10.1136/gut.47.suppl_4.iv54 PMC1766818

[b25] Gossen A. . Oxytocin plasma concentrations after single intranasal oxytocin administration - a study in healthy men. Neuropeptides 46, 211–215 (2012). 22884888 10.1016/j.npep.2012.07.001

[b26] Leake R. D., Weitzman R. E., Glatz T. H. & Fisher D. A. Plasma oxytocin concentrations in men, nonpregnant women, and pregnant women before and during spontaneous labor. J Clin Endocrinol Metab 53, 730–733 (1981). 7287862 10.1210/jcem-53-4-730

[b27] Leake R. D., Waters C. B., Rubin R. T., Buster J. E. & Fisher D. A. Oxytocin and prolactin responses in long-term breast-feeding. Obstet Gynecol 62, 565–568 (1983). 6684741

[b28] Carmichael M. S., Humbert R., Dixen J., Palmisano G., Greenleaf W. & Davidson J. M. Plasma oxytocin increases in the human sexual response. J Clin Endocrinol Metab 64, 27–31 (1987). 3782434 10.1210/jcem-64-1-27

[b29] Wang Y. L. . The interaction between the oxytocin and pain modulation in headache patients. Neuropeptides 47, 93–97 (2013). 23375440 10.1016/j.npep.2012.12.003

[b30] Yaksh T. L. . Preclinical toxicity screening of intrathecal oxytocin in rats and dogs. Anesthesiology 120, 951–961 (2014). 24492326 10.1097/ALN.0000000000000148 PMC5392224

[b31] Ohlsson B., Truedsson M., Djerf P. & Sundler F. Oxytocin is expressed throughout the human gastrointestinal tract. Regul Pept 135, 7–11 (2006). 16678285 10.1016/j.regpep.2006.03.008

[b32] Duridanova D. B., Nedelcheva M. D. & Gagov H. S. Oxytocin-induced changes in single cell K+ currents and smooth muscle contraction of guinea-pig gastric antrum. Eur J Endocrinol 136, 531–538 (1997). 9186274 10.1530/eje.0.1360531

[b33] Black L. V., Ness T. J. & Robbins M. T. Effects of oxytocin and prolactin on stress-induced bladder hypersensitivity in female rats. J Pain 10, 1065–1072 (2009). 19595642 10.1016/j.jpain.2009.04.007 PMC2757490

[b34] Reeta K., Mediratta P. K., Rathi N., Jain H., Chugh C. & Sharma K. K. Role of kappa- and delta-opioid receptors in the antinociceptive effect of oxytocin in formalin-induced pain response in mice. Regul Pept 135, 85–90 (2006). 16712978 10.1016/j.regpep.2006.04.004

[b35] Klooker T. K. . The mast cell stabiliser ketotifen decreases visceral hypersensitivity and improves intestinal symptoms in patients with irritable bowel syndrome. Gut 59, 1213–1221 (2010). 20650926 10.1136/gut.2010.213108

[b36] Wouters M. M., Vicario M. & Santos J. The role of mast cells in functional GI disorders. Gut (2015). 10.1136/gutjnl-2015-309151 26194403

[b37] Ohashi K., Sato Y., Iwata H., Kawai M. & Kurebayashi Y. Colonic mast cell infiltration in rats with TNBS-induced visceral hypersensitivity. J Vet Med Sci 69, 1223–1228 (2007). 18176016 10.1292/jvms.69.1223

[b38] Maurer M. . What is the physiological function of mast cells? Exp Dermatol 12, 886–910 (2003). 14719507 10.1111/j.0906-6705.2003.0109a.x

[b39] Abraham S. N. & St, John A. L. Mast cell-orchestrated immunity to pathogens. Nat Rev Immunol 10, 440–452 (2010). 20498670 10.1038/nri2782 PMC4469150

[b40] Cenac N. . Role for protease activity in visceral pain in irritable bowel syndrome. J Clin Invest 117, 636–647 (2007). 17304351 10.1172/JCI29255 PMC1794118

[b41] Pearce F. L., Befus A. D., Gauldie J. & Bienenstock J. Mucosal mast cells. II. Effects of anti-allergic compounds on histamine secretion by isolated intestinal mast cells. J Immunol 128, 2481–2486 (1982). 6176639

[b42] Bell A., Althaus M. & Diener M. Communication between mast cells and rat submucosal neurons. Pflugers Arch 467, 1809–1823 (2015). 25224285 10.1007/s00424-014-1609-9

[b43] Yue J. X. . Histamine upregulates Nav1.8 expression in primary afferent neurons via H2 receptors: involvement in neuropathic pain. CNS Neurosci Ther 20, 883–892 (2014). 24990156 10.1111/cns.12305 PMC6493056

[b44] Koibuchi Y., Ichikawa A., Nakagawa M. & Tomita K. Binding of active components of compound 48/80 to rat peritoneal mast cells. Eur J Pharmacol 115, 171–177 (1985). 2415370 10.1016/0014-2999(85)90688-0

[b45] Fu Y. S. . Pretreatment with Evans blue, a stimulator of BK channels, inhibits compound 48/80-induced shock, systemic inflammation, and mast cell degranulation in the rat. Histochem Cell Biol (2015). 10.1007/s00418-015-1332-4 26003544

[b46] Fowler C. J., Sandberg M. & Tiger G. Effects of water-soluble cigarette smoke extracts upon the release of beta-hexosaminidase from RBL-2H3 basophilic leukaemia cells in response to substance P, compound 48/80, concanavalin A and antigen stimulation. Inflamm Res 52, 461–469 (2003). 14652680 10.1007/s00011-003-1202-8

[b47] Ashmole I. & Bradding P. Ion channels regulating mast cell biology. Clin Exp Allergy 43, 491–502 (2013). 23600539 10.1111/cea.12043

[b48] Suzuki Y., Inoue T. & Ra C. L-type Ca^2+^ channels: a new player in the regulation of Ca^2+^ signaling, cell activation and cell survival in immune cells. Mol Immunol 47, 640–648 (2010). 19926136 10.1016/j.molimm.2009.10.013

[b49] Mousli M., Bronner C., Landry Y., Bockaert J. & Rouot B. Direct activation of GTP-binding regulatory proteins (G-proteins) by substance P and compound 48/80. Febs Lett 259, 260–262 (1990). 1688415 10.1016/0014-5793(90)80023-c

[b50] Kuno M., Okada T. & Shibata T. A patch-clamp study: secretagogue-induced currents in rat peritoneal mast cells. Am J Physiol 256, C560–C568 (1989). 2538065 10.1152/ajpcell.1989.256.3.C560

[b51] Aridor M., Traub L. M. & Sagi-Eisenberg R. Exocytosis in mast cells by basic secretagogues: evidence for direct activation of GTP-binding proteins. J Cell Biol 111, 909–917 (1990). 1697300 10.1083/jcb.111.3.909 PMC2116270

[b52] Bundoc V. G. & Keane-Myers A. IL-10 confers protection from mast cell degranulation in a mouse model of allergic conjunctivitis. Exp Eye Res 85, 575–579 (2007). 17765892 10.1016/j.exer.2007.07.005

[b53] Erkanli Senturk G. . The protective effect of oxytocin on ischemia/reperfusion injury in rat urinary bladder. Peptides 40, 82–88 (2013). 23262359 10.1016/j.peptides.2012.12.006

[b54] Csaba G. & Pallinger E. *In vitro* effect of hormones on the hormone content of rat peritoneal and thymic cells. Is there an endocrine network inside the immune system? Inflamm Res 56, 447–451 (2007). 18224286 10.1007/s00011-007-7021-6

[b55] Gilchrist M., McCauley S. D. & Befus, A.D. Expression, localization, and regulation of NOS in human mast cell lines: effects on leukotriene production. Blood 104, 462–469 (2004). 15044250 10.1182/blood-2003-08-2990

[b56] Kanwar S., Wallace J. L., Befus D. & Kubes P. Nitric oxide synthesis inhibition increases epithelial permeability via mast cells. Am J Physiol 266, G222–G229 (1994). 8141295 10.1152/ajpgi.1994.266.2.G222

[b57] Masini E., Salvemini D., Pistelli A., Mannaioni P. F. & Vane J. R. Rat mast cells synthesize a nitric oxide like-factor which modulates the release of histamine. Agents Actions 33, 61–63 (1991). 1716838 10.1007/BF01993127

[b58] Mannaioni P. F., Masini E., Pistelli A., Salvemini D. & Vane J. R. Mast cells as a source of superoxide anions and nitric oxide-like factor: relevance to histamine release. Int J Tissue React 13, 271–278 (1991). 1726322

[b59] Kurose I., Wolf R., Grisham M. B. & Granger D. N. Modulation of ischemia/reperfusion-induced microvascular dysfunction by nitric oxide. Circ Res 74, 376–382 (1994). 8118946 10.1161/01.res.74.3.376

[b60] Rettori V., Canteros G., Renoso R., Gimeno M. & McCann S. M. Oxytocin stimulates the release of luteinizing hormone-releasing hormone from medial basal hypothalamic explants by releasing nitric oxide. Proc Natl Acad Sci USA 94, 2741–2744 (1997). 9122267 10.1073/pnas.94.6.2741 PMC20160

[b61] Cattaneo M. G., Chini B. & Vicentini L. M. Oxytocin stimulates migration and invasion in human endothelial cells. Br J Pharmacol 153, 728–736 (2008). 18059319 10.1038/sj.bjp.0707609 PMC2259201

[b62] Yan Y., Zhao Z., Wan H., Wu R., Fang J. & Liu H. A novel fungus concentration-dependent rat model for acute invasive fungal rhinosinusitis: an experimental study. BMC Infect Dis 14, 3856 (2014). 25526739 10.1186/s12879-014-0713-y PMC4297382

[b63] Overman E. L., Rivier J. E. & Moeser A. J. CRF induces intestinal epithelial barrier injury via the release of mast cell proteases and TNF-alpha. PLoS One 7, e39935 (2012). 22768175 10.1371/journal.pone.0039935 PMC3386952

